# Cholesterol Metabolism in Cancer Patients: Mechanisms, Treatment-Related Effects, and Cardio-Oncology Management

**DOI:** 10.32604/or.2026.079215

**Published:** 2026-09-14

**Authors:** Mariagrazia Piscione, Barbara Pala, Francesco Cribari, Paola Gualtieri, Dario Gaudio, Marco Alfonso Perrone, Laura Di Renzo

**Affiliations:** 1 Santissima Annuziata Hospital, Via dei Vestini, Chieti, Italy; 2 PhD School of Applied Medical-surgical sciences, Tor Vergata University of Rome, Via Montpellier 1, Rome, Italy; 3 UOC Cardiologia, Ospedale IDI-IRCCS, Rome, Italy; 4 Section of Clinical Nutrition and Nutrigenomics, Department of Biomedicine and Prevention, Tor Vergata University of Rome, Via Montpellier 1, Rome, Italy; 5 Fondazione Policlinico Campus Bio-Medico, University of Rome, Via Alvaro del Portillo 200, Rome, Italy; 6 Division of Cardiology, Tor Vergata University of Rome, Via Montpellier 1, Rome, Italy

**Keywords:** Cholesterol metabolism, dyslipidaemia, cancer progression, cardio-oncology, lipid-lowering therapies

## Abstract

Cholesterol metabolism is central to cancer biology, influencing tumour initiation, progression, and therapeutic response, while contributing to the increased cardiovascular risk observed in cancer patients. Epidemiological studies investigating the relationship between circulating cholesterol levels and cancer risk have yielded conflicting results, reflecting substantial biological heterogeneity, tumour-specific metabolic demands, and methodological biases such as reverse causality. At the cellular level, malignant cells exhibit elevated cholesterol uptake and synthesis to sustain membrane biogenesis, lipid raft-dependent oncogenic signalling, and rapid proliferation. Cholesterol and its oxidized derivatives further modulate inflammation, angiogenesis, immune evasion, and key signalling pathways. Anticancer therapies profoundly disrupt lipid homeostasis; conventional chemotherapies, targeted therapies, hormone-modulating agents, and immunotherapies can induce dyslipidaemia and accelerate atherosclerotic disease, thereby contributing to long-term morbidity in cancer survivors. Conversely, lipid-lowering therapies—particularly statins—have emerged as pivotal tools in cardio-oncology, primarily for cardiovascular protection. Beyond this role, growing evidence suggests potential adjunctive antitumor effects mediated through inhibition of the mevalonate pathway, blockade of oncogenic signalling, and modulation of the tumour microenvironment. Novel agents, including ezetimibe, bempedoic acid, and proprotein convertase subtilisin-kexin type 9 inhibitors, provide additional therapeutic options, with emerging evidence supporting their immunomodulatory and anticancer properties, especially in combination with immune checkpoint inhibitors. Cholesterol metabolism plays a critical role in both CV disease and cancer biology, with growing evidence suggesting a complex bidirectional relationship between lipid homeostasis and tumour development. In addition, several anticancer therapies may profoundly affect lipid metabolism, thereby contributing to CV risk in cancer patients. The aim of this review is to provide a comprehensive overview of the interplay between cholesterol metabolism and cancer, to summarize the lipid-modifying effects of anticancer therapies, and to discuss the potential role of lipid-lowering strategies within the evolving framework of cardio-oncology care.

## 1 Introduction

Advances in cancer diagnosis and treatment have led to a growing population of long-term cancer survivors, in whom cardiovascular (CV) disease has emerged as a leading cause of morbidity and mortality [1]. Among the many determinants of CV risk, altered cholesterol metabolism has a central position, yet its role in oncology extends far beyond traditional atherosclerotic disease [1]. Cholesterol is not only a structural component of cellular membranes, but also a key modulator of membrane microdomains, signal transduction, steroid hormone synthesis, and immune cell function [1]. All these processes are critically involved in tumour initiation, progression, and therapeutic resistance [2].

Experimental data demonstrate that many cancer cells reprogram cholesterol metabolism by upregulating endogenous synthesis, increasing low-density lipoprotein (LDL) uptake, and altering intracellular trafficking [2]. These adaptations support rapid proliferation, invasive behaviour, and resistance to cell death [3]. At the same time, systemic cholesterol levels in cancer patients are influenced by tumour burden, chronic inflammation, cachexia, and the direct effects of anticancer therapies, resulting in a complex and often dynamic lipid profile [4]. This systemic-tumoral interplay is particularly relevant for patients treated with potentially cardiotoxic therapies, in whom baseline dyslipidaemia and treatment-induced lipid changes may synergistically increase CV risk [4].

Beyond their established CV benefits, cholesterol-lowering agents—especially statins—have attracted considerable interest as potential anticancer therapies. As a matter of fact, preclinical studies have shown that statins and other modulators of cholesterol homeostasis can interfere with oncogenic signalling pathways, impair membrane-dependent receptor function, and modulate the tumour immune microenvironment [5]. Observational clinical studies have suggested associations between statin use and improved cancer outcomes in certain tumour types, although robust causal evidence remains limited and confounded by indication bias and heterogeneity in study design [5].

To summarize, cholesterol metabolism plays a central role in both CV disease and cancer biology, and increasing evidence suggests a complex bidirectional relationship between lipid homeostasis and tumour development [1]. In addition, both malignancy itself and several anticancer therapies may promote pro-atherogenic alterations in lipid metabolism, thereby increasing CV risk in cancer patients [4]. In this context, an important issue is whether emerging lipid-lowering agents and novel metabolic modulators may help support cancer patients by mitigating the pro-atherogenic effects of the disease itself while also counteracting the cardiometabolic toxicity associated with anticancer therapies. This review aims to explore this possibility and to discuss the potential role of lipid-modulating strategies in the integrated management of cancer- and therapy-related CV risk.

## 2 Literature Search Strategy and Article Selection

The literature search for this narrative review was conducted using the PubMed database. Articles published between 2017 and 2025 were considered. The search strategy was based on combinations of the following keywords: *“chemotherapy and lipid metabolism”, “immunotherapy and lipid metabolism”, “chemotherapy and dyslipidaemia”, “lipid-lowering drugs and dyslipidaemia”.*

Eligible articles were selected based on their relevance to the role of statins and other lipid-lowering therapies in cancer patients, with particular attention to CV management, cardiotoxicity prevention, and potential antitumour effects. Both preclinical and clinical studies were included, encompassing observational studies, randomized and non-randomized clinical trials, experimental studies, and relevant narrative and systematic reviews. Titles and abstracts were independently screened by the authors to assess eligibility. Full-text articles of potentially relevant studies were subsequently reviewed, and those deemed most pertinent to the scope of the review were included. Any discrepancies in study selection were resolved through consensus among the authors.

Given the narrative nature of this review, no formal quality assessment or risk-of-bias scoring system was applied.

## 3 Basics of Cholesterol Metabolism in Health

### 3.1 Cholesterol Homeostasis

**Figure 1:**
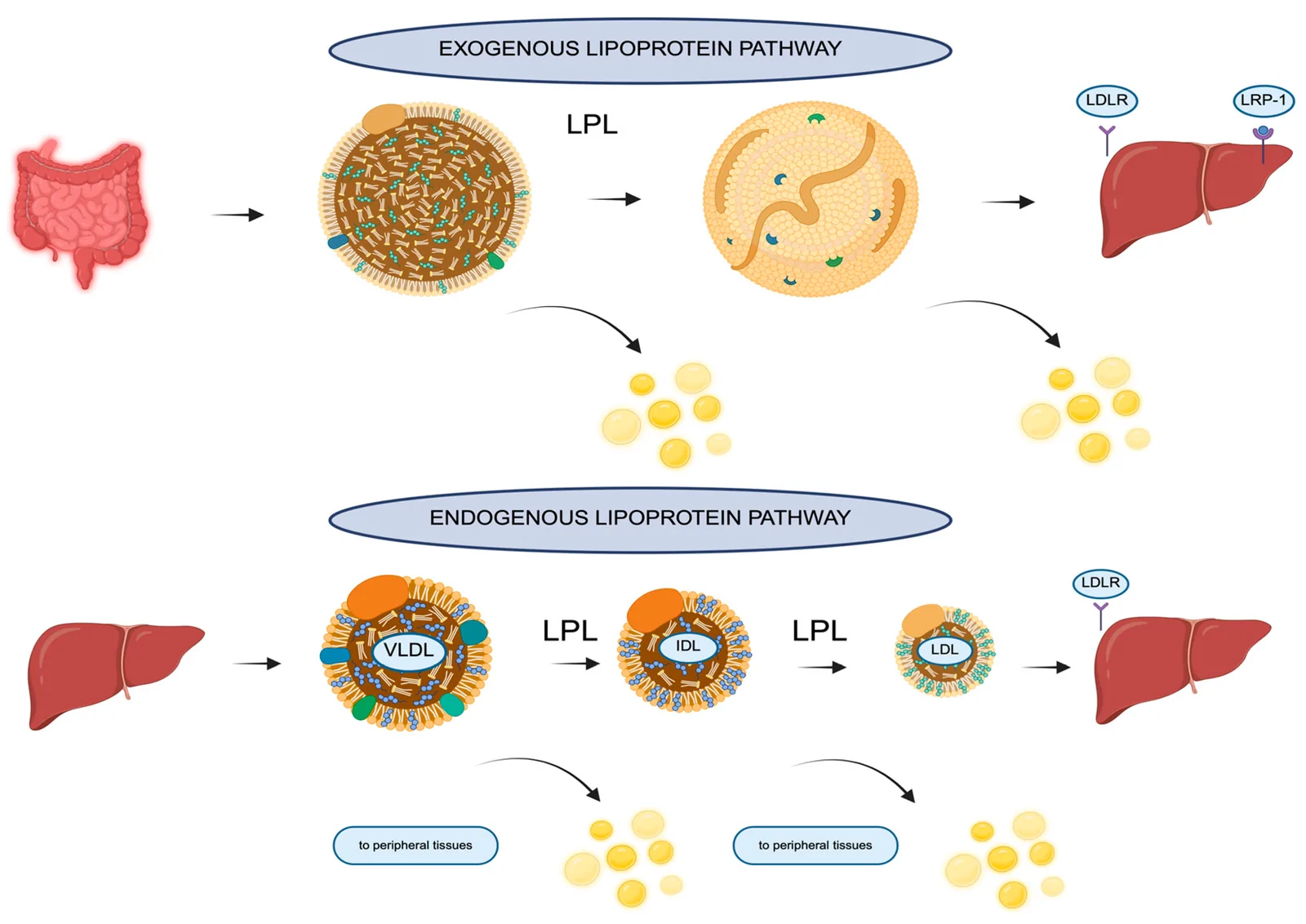
Endogenous and exogenous lipoprotein pathways (Created in BioRender.com). Cholesterol present in the intestinal lumen is absorbed by enterocytes and secreted into the circulation within chylomicrons. In peripheral tissues, triglyceride hydrolysis by lipoprotein lipase generates chylomicron remnants, which are taken up by hepatocytes via low-density lipoprotein receptor (LDLR)- and LDL receptor-related protein 1 (LRP1)-mediated endocytosis. In parallel, hepatocytes synthesize and secrete very-low-density lipoproteins (VLDL), which undergo progressive triglyceride removal by tissue LPL to form intermediate-density lipoproteins (IDL) and subsequently low-density lipoproteins (LDL). Circulating LDL particles deliver cholesterol and triglycerides to peripheral tissues and are ultimately cleared from the circulation through LDLR-mediated hepatic endocytosis.

In healthy physiology, cholesterol homeostasis reflects a highly coordinated balance between intestinal absorption, endogenous hepatic synthesis, lipoprotein transport, and receptor-mediated cellular uptake [1]. Two major pathways contribute to the cholesterol pool of the body: the exogenous pathway, which derives from intestinal absorption, and the endogenous pathway, driven by hepatic biosynthesis [6] [Fig. 1].

### 3.2 Exogenous Cholesterol Pathway

Cholesterol entering the intestinal lumen originates from dietary intake, biliary secretion, and turnover of intestinal epithelial cells [6]. Absorption by enterocytes is mediated by the Niemann-Pick C1-like 1 (NPC1L1) transporter, after which cholesterol is incorporated into chylomicrons (CMs) and released into the lymphatic system [6]. As CMs circulate, peripheral lipoprotein lipase (LPL) hydrolyses triglycerides (TG), generating CM remnants that are taken up by hepatocytes predominantly through LDL receptor (LDLR)-dependent endocytosis [6]. This exogenous route delivers dietary fatty acids to peripheral tissues and returns cholesterol to the liver for repackaging into very-low-density lipoproteins (VLDL) or for conversion into bile acids [7].

### 3.3 Endogenous Cholesterol Synthesis

Endogenous cholesterol synthesis occurs via the mevalonate pathway, beginning with the production of acetyl-CoA from citrate through ATP-citrate lyase. Acetyl-CoA is progressively converted into cholesterol through a series of enzymatic reactions, the rate-limiting step being the reduction of 3-hydroxy-3-methylglutaryl-coenzyme A (HMG-CoA) to mevalonate, catalysed by HMG-CoA reductase (HMG-CoAR), the molecular target of statins [8]. Newly synthesized cholesterol is secreted in VLDL, which progressively loses TG through LPL activity to form intermediate-density lipoproteins (IDL) and ultimately LDL [8]. Approximately 70% of circulating LDL particles are cleared by hepatocytes via LDLR-mediated endocytosis, with the remainder taken up by extrahepatic tissues [9]. Increased LDLR expression enhances LDL clearance, whereas reduced receptor availability leads to elevated plasma LDL levels [9].

### 3.4 Intracellular Cholesterol Sensing

The intracellular cholesterol pool is tightly regulated by the sterol regulatory element-binding protein 2 (SREBP-2) pathway, which functions as a sensor of hepatocellular cholesterol content [9]. When cholesterol levels are sufficient, SREBP-2 remains sequestered in the endoplasmic reticulum [9]. Cholesterol depletion releases SREBP-2, allowing proteolytic activation and nuclear translocation, where it upregulates transcription of genes encoding HMG-CoAR, LDLR, and NPC1L1. SREBP-2 also induces proprotein convertase subtilisin/kexin type 9 (PCSK9), a serine protease that promotes LDLR internalization and degradation, acting as a homeostatic counter-regulator to prevent excessive receptor activity [9].

### 3.5 Cholesterol Efflux and LXR Signalling

A complementary regulatory axis is provided by the liver X receptors (LXRs), nuclear receptors activated by oxysterols—oxidized derivatives of cholesterol. In peripheral tissues, LXR activation induces the cholesterol efflux transporters ATP binding cassette transporter A1 (ABCA1) and ATP binding cassette transporter G1 (ABCG1), facilitating reverse cholesterol transport to high-density lipoprotein (HDL) [10]. In the liver, LXRs upregulate ATP-binding cassette transporter G5 (ABCG5) and ATP-binding cassette transporter G8 (ABCG8), enhancing biliary excretion of cholesterol, while also promoting lipogenesis and downregulating LDLR expression [10]. LXRs further modulate inflammatory responses, particularly in macrophages, where ABCA1-mediated efflux prevents foam-cell formation and supports an anti-inflammatory phenotype [10].

Together, these interconnected pathways maintain cholesterol homeostasis under physiological conditions and form the biological foundation upon which cancer-related alterations in lipid metabolism are superimposed [10].

## 4 Cancer-Associated Dysregulation of Cholesterol Metabolism

### 4.1 Cholesterol-Cancer Paradox

**Figure 2:**
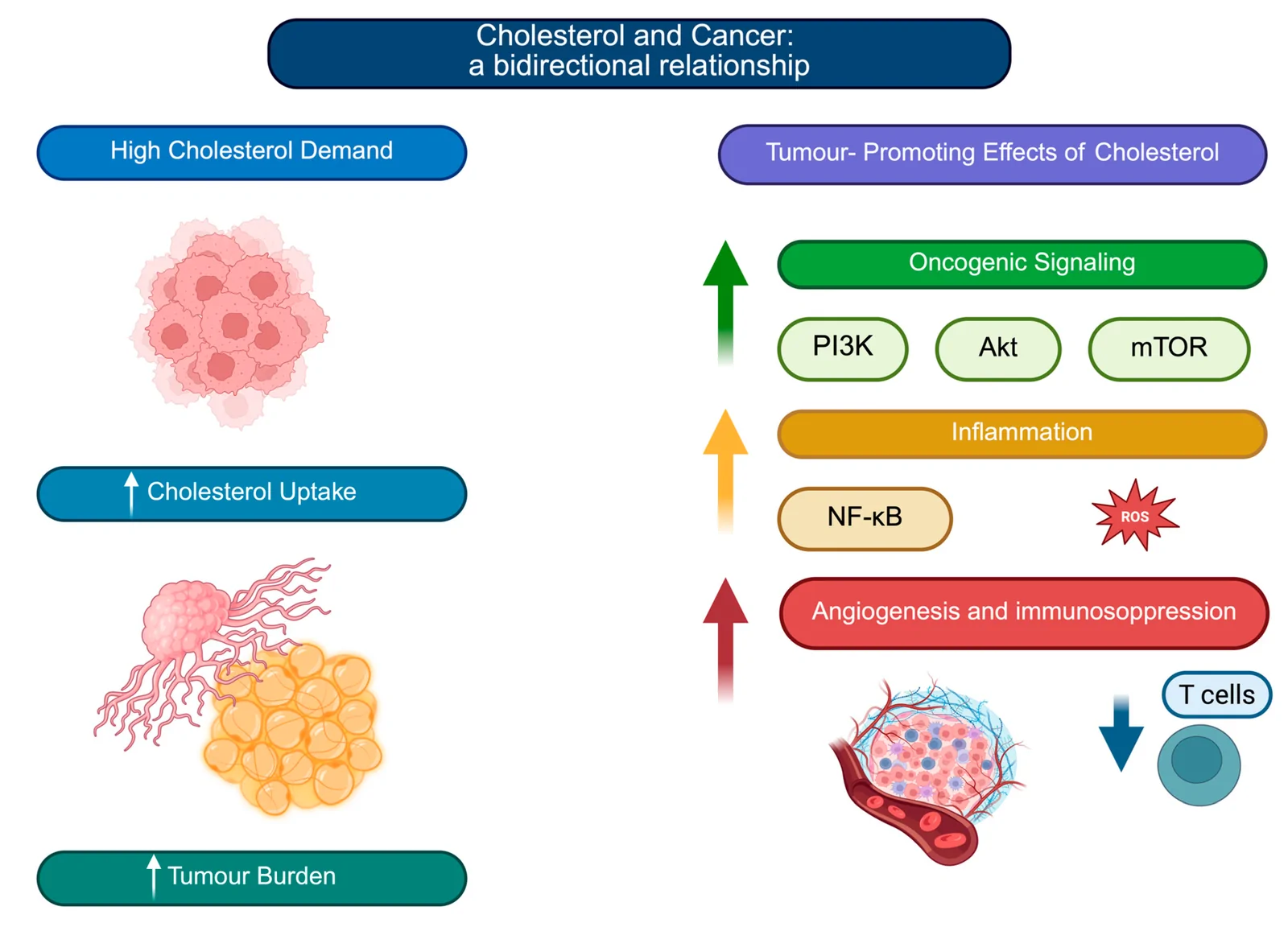
Cholesterol-cancer interplay: a bidirectional and context-dependent relationship (Created in BioRender.com). This schematic illustrates the bidirectional relationship between cholesterol metabolism and cancer. On the left, increased tumour cholesterol demand drives enhanced cholesterol uptake, lipid raft formation, and membrane biogenesis, supporting tumour growth, invasiveness, and therapy resistance while potentially lowering circulating cholesterol levels despite increasing tumour burden. On the right, oncogenic signalling pathways promote *de novo* cholesterol synthesis, leading to hypercholesterolemia and reinforcing tumour progression. Elevated cholesterol further sustains cancer development by activating oncogenic signalling cascades (PI3K-AKT-mTOR), promoting inflammation (NF-κB), angiogenesis, and immune evasion through suppression of anti-tumour T-cell responses and enrichment of tumour-associated macrophages. Abb: PI3K: Phosphatidylinositol 3-kinase, AKT: protein kinase B, mTOR: Mechanistic Target of Rapamycin Kinase.

The relationship between systemic cholesterol levels and cancer incidence or progression is far from linear and remains the subject of extensive scientific debate [11]. Dyslipidaemia may represent both a cause and a consequence of cancer, with directionality critically depending on tumour type, stage, and metabolic phenotype [12]. Indeed, although cholesterol is an essential structural component of cellular membranes and a critical substrate for cell proliferation, available epidemiologic data reveal conflicting associations across cancer types [11] [Fig. 2].

Elevated plasma cholesterol has been linked to an increased risk of several solid tumours, including colorectal, breast, prostate, and testicular cancers; however, this association is not consistently observed across malignancies [12]. Multiple physiopathologic explanations have been proposed [12]. On one hand, malignant cells exhibit a markedly increased demand for cholesterol to sustain membrane biogenesis, lipid raft formation, oncogenic signalling, and rapid clonal expansion [12]. Enhanced cholesterol uptake by tumour cells may therefore reduce circulating cholesterol levels, resulting in lower serum concentrations despite increased tumour burden [12]. On the other hand, hypercholesterolemia in patients with cancer may reflect upregulated endogenous cholesterol synthesis driven by activated oncogenic pathways, generating an apparent association that is mechanistically reversed [12]. 

### 4.2 Biological Heterogeneity

This complexity is exemplified by a large U.S. observational cohort of more than 17,050 individuals, which has demonstrated a substantially higher prevalence of dyslipidaemia among patients with cancer compared with non-cancer controls (53% vs. 32%) [13]. However, such epidemiologic associations must be interpreted with caution, as they likely obscure considerable biological heterogeneity. Cholesterol homeostasis is inherently tissue-specific [12]. Tumours arising in organs with high basal sterol turnover—such as the liver, intestine, or steroidogenic tissues—display metabolic requirements that differ fundamentally from those originating in low-turnover environments [12]. Dietary cholesterol intake further contributes to interindividual variability and may influence tumorigenesis through alterations in bile acid metabolism, gut microbial composition, and systemic inflammatory tone [12].

### 4.3 Conflicting Associations

These divergent biological contexts are reflected in the inconsistencies of epidemiologic data. A prospective Korean cohort study has demonstrated that elevated total cholesterol (TC) (≥240 mg/dL) is associated with an increased incidence of prostate and colorectal cancers in men and breast cancer in women, whereas higher cholesterol levels were inversely associated with the risk of liver and gastric cancers in both sexes and lung cancer in men [14]. Similarly, while some studies have linked elevated LDL cholesterol or TG levels with increased cancer risk [15], others report inverse associations, such as reduced risks of gastric [16] or oral cancers [17]. These findings underscore that lipid-cancer relationships are not universally directional but instead context dependent, influenced by tumour biology and metabolic phenotype.

### 4.4 Reverse Causality

Additional complexity arises from the temporal relationship between lipid measurements and cancer diagnosis [18]. Several studies have reported a short-term inverse association between TC or HDL levels and cancers diagnosed shortly after baseline lipid assessment, an association that disappears with longer follow-up durations [18]. This pattern suggests reverse causality, whereby early, subclinical cancer lowers circulating lipid levels, creating the illusion of a protective effect [18]. The prospective findings of Strasak et al. support this interpretation. In their 19-year cohort study of 172,210 healthy Austrian adults, individuals in the highest tertile of total serum cholesterol exhibit a reduced short-term risk of overall cancer and of malignancies affecting the digestive and lymphohematopoietic systems compared with those in the lowest tertile [18]. This apparent protective association disappears when analyses are restricted to cancers diagnosed more than 5, 12, or 24 months after baseline, reinforcing the role of tumour-related metabolic alterations rather than cholesterol itself [18].

### 4.5 Oncogenic Effects of Cholesterol

From a mechanistic perspective, hypercholesterolemia exerts pleiotropic pro-tumorigenic effects [19]. Elevated cholesterol enhances oxidative stress and ROS formation, promoting deoxyribonucleic acid (DNA) damage and genomic instability [19]. It fuels a chronic inflammatory state through activation of nuclear factor kappa-light-chain-enhancer of activated B cells (NF-κB) and inflammasome pathways, supports endothelial proliferation and angiogenesis, and fosters an immunosuppressive microenvironment characterized by dysfunctional T-cell responses and increased recruitment of tumour-associated macrophages [19]. In parallel, cholesterol serves as an indirect modulator through effects on membrane organization, lipid rafts and receptor signaling of oncogenic signalling cascades—including Hedgehog, phosphatidylinositol 3-kinase (PI3K), protein kinase B (AKT), mechanistic target of rapamycin (mTOR), and wingless-related integration site (Wnt)/β-catenin—each of which contributes to tumour survival, growth, and therapy resistance [20]. These pathways can, in turn, upregulate SREBP-2, the master transcriptional regulator of cholesterol synthesis, thereby amplifying intracellular sterol accumulation and closing a vicious cycle promoting tumour expansion [20].

### 4.6 Oxysterol-LXR Axis

Oxysterols and the liver X receptor axis add yet another level of mechanistic nuance [21]. The role of LXR signalling in cancer biology is dualistic and highly context-dependent [21]. In hormone-responsive tissues, such as the breast, 27-hydroxycholesterol (27HC)—a cholesterol metabolite acting as both an oestrogen receptor ligand and an LXR agonist—promotes tumour growth, metastasis, and endocrine therapy resistance, establishing a mechanistic bridge between hypercholesterolemia and breast cancer risk [21]. Conversely, in other malignancies such as lung, pancreatic, hepatocellular, and colorectal cancers, LXR activation has been shown to inhibit proliferation, induce apoptosis, or suppress metastatic potential by modulating lipid efflux, inflammatory gene expression, and metabolic fitness [21]. These divergent effects highlight the importance of tumour-specific metabolic wiring, microenvironmental cues. Differential engagement of LXRα versus LXRβ isoforms likely contributes to this context dependency. A deeper understanding of how oxysterol-LXR interactions reprogram cancer cell biology may unveil new therapeutic opportunities, particularly in the form of selective LXR modulators or agents targeting sterol intermediates [21].

Taken together, the interplay between cholesterol metabolism and cancer includes a complex network of systemic, cellular, and molecular determinants. Knowing these mechanisms has profound implications not only for clarifying disease pathophysiology but also for informing preventive strategies, identifying metabolic vulnerabilities, and optimizing the use of lipid-lowering therapies in oncology practice.

### 4.7 Oxidized Low-Density Lipoproteins and Cancer

Oxidized low-density lipoproteins (oxLDL) have emerged as biologically active mediators at the intersection of dyslipidaemia, cancer progression, and cardiovascular toxicity [22]. Elevated circulating levels of LDL and oxLDL, together with overexpression of their cognate receptors—including the LDLR, lectin-like oxidized LDL receptor-1 (LOX-1), and cluster of differentiation 36 (CD36)—have been associated with tumour proliferation, invasion, angiogenesis, and metastatic potential across multiple malignancies. LOX-1 overexpression has been consistently linked to aggressive tumour phenotypes and poor prognosis, notably in colorectal, breast, and lung cancers [22]. More in detail, oxLDL promote cancer progression by activating pro-inflammatory and pro-oxidative signalling cascades, inducing endothelial-to-mesenchymal transition, and modulating autophagic flux, thereby facilitating tumour cell survival, vascular remodelling, and metastatic dissemination [22].

## 5 Anticancer Therapies with the Potential to Induce Lipid Profile Alterations

Cancer and atherosclerotic cardiovascular disease (ASCVD) share several classical risk factors, including age, smoking, and diabetes. However, growing evidence indicates that individuals with a history of cancer have an intrinsically higher risk of developing ASCVD, independent of traditional risk factors [22]. This observation suggests that malignancy itself, together with chronic inflammation, metabolic reprogramming, and the cardiotoxic effects of anticancer therapies, contributes to long-term vascular injury and altered lipid handling [22]. Many oncologic treatments—chemotherapy, targeted therapies, immunotherapy—can profoundly affect lipid homeostasis and accelerate atherosclerotic processes [22,23] [Table 1]. 

### 5.1 Anthracyclines

Anthracyclines are among the most widely used chemotherapeutic agents to treat solid and hematologic malignancies [24]. Their antitumour efficacy derives primarily from topoisomerase II inhibition, mitochondrial dysfunction, and generation of reactive oxygen species (ROS), ultimately impairing DNA and ribonucleic acid (RNA) synthesis and promoting cell death [25]. While their dose-dependent cardiomyopathy remains the most recognized toxicity, emerging evidence demonstrates that anthracyclines also exert significant effects on lipid metabolism, potentially contributing to accelerated ASCVD in cancer survivors [26,27]. 

Recent studies indicate that these drugs interfere with cholesterol efflux pathways, notably by reducing the activity of LXR-α and peroxisome proliferator-activated receptor γ (PPAR-γ), key transcriptional regulators of ATP binding cassette transporter A1 (ABCA1)-mediated reverse cholesterol transport [27]. Sharma et al. systematically evaluate the *in vivo* metabolic effects of doxorubicin, epirubicin, and related agents in human hepatocyte models, demonstrating a marked reduction in ABCA1 mRNA expression and a 20–30% dose-dependent decrease in cholesterol efflux, with similar findings for epirubicin [28]. Complementary work in doxorubicin-treated myocytes and murine hearts shows elevated intracellular cholesterol and precursor sterols, reinforcing the concept that anthracyclines impair cholesterol handling at the cellular level [29].

Paradoxically, these drugs are also shown to suppress HMGCR activity—the rate-limiting enzyme in cholesterol synthesis—which would theoretically reduce circulating LDL [30]. However, clinical observations consistently document increased apolipoprotein B (apo B), a structural protein of atherogenic lipoproteins, suggesting that anthracycline-induced metabolic disruption extends beyond isolated enzymatic effects [31].

Clinical datasets further support these insights. Two large studies by Lu et al. and He et al., including more than 1100 patients undergoing anthracycline-predominant chemotherapy, demonstrate consistent increases in TC, LDL cholesterol, and TG at treatment completion [25,26]. Effects on HDL are less uniform but tend toward reduction, consistent with experimental suppression of ABCA1-mediated efflux. Long-term data remain limited, yet findings by Arpino et al. In 433 women with early breast cancer revealed persistent elevations in TC, LDL, and TG up to 24 months after anthracycline/cyclophosphamide followed by taxanes, suggesting a durable impairment of lipid homeostasis.

Taken together, evidence from molecular, cellular, animal, and human studies suggests that anthracyclines promote a dyslipidaemic phenotype. This latter is characterized by impaired cholesterol efflux, elevated atherogenic lipoproteins, and potentially long-lasting metabolic alterations. Although further investigation is needed to define causality and long-term cardiovascular impact, current evidence supports the incorporation of lipid monitoring and ASCVD-preventive strategies in patients treated with anthracyclines [31].

### 5.2 Taxanes

Taxanes exert their antitumour activity primarily by stabilizing guanosine diphosphate (GDP)-bound tubulin within microtubules, thereby impairing their dynamic function and ultimately inhibiting mitotic progression [32]. These agents are widely used across multiple solid malignancies and represent a fundamental component of breast cancer chemotherapy [32]. Beyond their cytotoxic role, emerging evidence indicates that taxanes may also influence lipid homeostasis [32].

In a proteomic study involving 80 women receiving paclitaxel, even a single infusion altered the expression of more than 180 proteins, many of which are integral to lipid metabolism—particularly pathways involved in lipoprotein synthesis, transport, and clearance [33]. Complementing these observations. Demonstrated that paclitaxel increases HMG-CoAR activity in human hepatocytes, a mechanism that could enhance endogenous cholesterol synthesis [33]. Paclitaxel has also been associated with reduced apo B levels and downregulation of LDL receptor expression, changes that may impair LDL clearance and predispose to dyslipidaemia [31].

Taxanes are rarely administered as monotherapy, which complicates the isolation of their individual metabolic effects [32]. Most clinical regimens combine taxanes with anthracyclines, and thus their dyslipidaemic profile often reflects synergistic or additive pharmacologic interactions [32]. Nevertheless, available evidence suggests a measurable independent impact [32]. In a subgroup analysis comparing patients treated with and without taxanes, He et al. reported that taxane-containing regimens were associated with a more pronounced increase in lipid abnormalities [26]. Additional studies have corroborated that regimens incorporating taxanes—particularly when paired with anthracyclines—tend to produce higher rates of treatment-associated dyslipidaemia [26,27,31].

Together, these findings support the concept that taxanes may contribute to alterations in lipid metabolism through multiple mechanisms—including increased cholesterol synthesis and reduced LDL clearance—although further dedicated studies are needed to precisely define their isolated metabolic effects.

### 5.3 Tyrosine Kinase Inhibitors

Tyrosine kinase inhibitors (TKIs) are integral to the treatment landscape of gastrointestinal, genitourinary, hematologic, and thoracic malignancies [34]. Although primarily designed to inhibit oncogenic signaling cascades—most notably those driving tumour proliferation and angiogenesis—TKIs exert a spectrum of off-target effects that include alterations in lipid metabolism and potentially cardiotoxic consequences [34,35].

Imatinib, the prototypical first-generation TKI targeting breakpoint cluster region abelson murine leukemia viral oncogene homolog 1 (BCR-ABL1), illustrates this metabolic complexity [34]. Beyond its antileukemic activity, imatinib modulates lipid pathways by reducing cytoplasmic phosphorylation of LDL receptor-related protein, a regulator of LDL trafficking, lysosomal enzyme activation, and cholesterol homeostasis [34]. Preclinical work supports these effects: in hypercholesterolemic rabbits, imatinib not only lowered serum cholesterol levels but also attenuated the vascular and hepatic toxicity associated with diet-induced hyperlipidaemia [34]. These benefits correlated with reductions in inflammatory markers such as C-reactive protein and improvements in hepatic enzyme profiles, indicating that imatinib exerts multifaceted anti-atherosclerotic actions [35]. However, the metabolic impact of TKIs is far from uniform. Agents directed at different molecular pathways may induce opposite or synergistic effects on lipid regulation [34]. Some TKIs have been linked to dyslipidaemia, endothelial dysfunction, or acceleration of atherosclerotic processes, suggesting that the net lipid effect depends on the specific kinase inhibited, tissue distribution, and interaction with host metabolic pathways [34,35].

Collectively, these findings underscore the heterogeneity of TKI-induced metabolic alterations and highlight the need for individualized lipid monitoring in patients receiving these therapies, particularly in those with pre-existing CV risk factors.

### 5.4 Mechanistic Target of Rapamycin Inhibitors

The mechanistic Target of Rapamycin (mTOR) pathway plays a central role in regulating gene transcription, protein synthesis, cell growth, and immune cell differentiation [36]. Although traditionally used as immunosuppressive agents in solid organ transplantation, mTOR inhibitors—including everolimus, temsirolimus, and deforolimus—also serve as targeted therapies in various malignancies [36]. Their interference with lipid metabolism is well documented and represents a clinically relevant off-target effect [36]. mTOR inhibition leads to dyslipidaemia primarily through two mechanisms: reduced expression of lipogenic enzymes, which affects TG handling, and downregulation of LDL receptors, which impairs LDL clearance from the circulation [37]. In a systematic review of 17 randomized controlled trials in kidney transplant recipients, Kasiske et al. found that 14 studies reported significant elevations in TC and/or TG in patients treated with mTOR inhibitors compared with control groups [38]. Notably, these increases frequently prompted the initiation of lipid-lowering therapy, underscoring the clinical burden of mTOR-related metabolic disturbances [38]. Importantly, transplant populations typically receive lower drug doses than those used in oncology, suggesting that dyslipidaemic effects in cancer patients may be even more pronounced [38].

Dose-dependent lipid alterations have also been observed in early-phase oncology trials. In a phase I study of deforolimus for advanced malignancies, TC increased by an average of 7.4 mg/dL for every 10 mg dose increment, up to the maximum tested dose of 75 mg [38]. Similarly, in a phase III trial of temsirolimus for metastatic renal-cell carcinoma, hypercholesterolaemia occurred in 24% of patients, compared with only 4% in the interferon-α control arm [39].

The clinical implications of mTOR inhibitor-induced dyslipidaemia remain uncertain. Paradoxically, mTOR inhibition may attenuate pathways involved in atherogenesis, making it difficult to determine whether observed lipid elevations translate into increased CV risk [40]. Furthermore, observational data suggesting reduced CV events among patients treated with mTOR inhibitors are confounded by the higher use of statins and other lipid-lowering therapies in response to drug-induced hyperlipidaemia [41,42].

Overall, while mTOR inhibitors clearly perturb lipid metabolism, their net cardiovascular impact remains unresolved, and dedicated studies are needed to clarify their long-term effects on atherosclerotic disease in oncology populations.

### 5.5 Alkylating Agents

Alkylating agents exert their antineoplastic effect primarily through the formation of covalent cross-links between DNA and RNA strands, ultimately impairing replication and halting cell division [43]. Among this class, cyclophosphamide is the most widely used and is well known for its potential to induce fulminant cardiotoxicity at high doses [43]. However, its impact on lipid metabolism remains far less clearly defined [43].

Experimental data suggest a limited direct effect on lipogenesis. *In vitro* exposure of human hepatocytes to cyclophosphamide did not significantly modify key metabolic pathways regulating lipid synthesis or efflux [38]. Animal studies, however, have yielded mixed results: toxic, high-dose administration has been associated with dyslipidaemia in rat models, whereas low-dose cyclophosphamide reduced atherosclerotic progression in mice, suggesting potential dose-dependent divergence in metabolic impact [44,45].

Human clinical data are similarly nuanced. Regimens containing cyclophosphamide in the absence of anthracyclines generally do not alter lipid profiles and may even improve LDL concentrations [45]. This contrasts with the well-documented dyslipidaemic effects observed when cyclophosphamide is administered alongside anthracyclines, complicating the ability to attribute metabolic changes to a single agent [44]. Overall, current evidence indicates that alkylating agents—particularly cyclophosphamide—do not independently induce clinically significant dyslipidaemia at conventional therapeutic doses [44]. Nonetheless, the paucity of data regarding other alkylating compounds, combined with conflicting preclinical observations, underscores the need for more systematic investigation.

### 5.6 Platinums

Platinum complexes consist of a positively charged platinum center coordinated to anionic ligands, enabling them to form DNA cross-links that inhibit transcription and protein synthesis, ultimately inducing tumour cell death [46]. Although cholesterol metabolism appears to influence the antitumour efficacy of platinum agents [47], evidence linking these drugs to clinically meaningful alterations in lipid homeostasis is limited.

Preclinical studies provide some preliminary signals. In a rat model, Najam et al. demonstrated that both cisplatin and oxaliplatin were associated with increased LDL and TG concentrations 30 days after treatment discontinuation compared with saline-injected controls [48]. Histologic evaluation also revealed myofibrillar loss and vascular wall thickening in cisplatin-treated animals, findings not observed with oxaliplatin; however, these morphological changes have not been validated in humans [48].

Human data remain sparse and inconsistent. One adjuvant combination regimen incorporating cisplatin, carboplatin, and nedaplatin was associated with increases in TC, LDL, HDL, and TG at the end of treatment [49]. Yet other clinical studies found no significant changes in plasma cholesterol, either in the immediate post-treatment period or after long-term follow-up extending to five years [48,49].

Overall, current evidence suggests that platinum compounds are not consistently associated with dyslipidaemia, and any observed effects may be transient, regimen-specific, or confounded by concomitant therapies.

### 5.7 Antimetabolites

Antimetabolites comprise a heterogeneous class of purine and pyrimidine analogues that disrupt DNA synthesis by becoming incorporated into nucleic acid chains, thereby inhibiting tumour cell proliferation [50]. Despite their widespread use across multiple malignancies, their effects on lipid metabolism remain variably characterized. 

Preclinical studies have suggested potential lipid-lowering properties. In a rabbit model, 5-fluorouracil (5-FU) was associated with reduced cholesterol levels, although this observation has not been reproduced in humans [51]. Methotrexate may also modulate lipid balance through upregulation of ABCA1 and 27-hydroxylase—enzymes involved in cholesterol efflux—resulting in decreased cholesterol levels [52]. This phenomenon has been described in patients with rheumatoid arthritis receiving chronic methotrexate therapy [52]. Nevertheless, in the Cardiovascular Inflammation Reduction Trial, low-dose methotrexate produced only minimal reductions in LDL, TG, and HDL, without translating into measurable CV benefit [53].

In oncology populations, findings appear more heterogeneous. Among patients with colorectal cancer treated with fluoropyrimidine-based regimens (5-FU or capecitabine), increases in TC, HDL, and TG were observed at the end of treatment, while LDL levels paradoxically decreased [54]. Capecitabine monotherapy has also been rarely implicated in cases of severe hypertriglyceridaemia [55].

For the broader class of antimetabolites, no consistent association with dyslipidaemia has been established to date. Overall, the impact of these agents on lipid metabolism—and consequently on ASCVD risk—remains uncertain and likely varies depending on drug, dose, treatment duration, and patient-specific factors.

### 5.8 Hormone-Targeting Therapies

Sex hormones play a central role in the regulation of cholesterol synthesis, transport, and efflux [56]. Estrogen suppresses hepatic HMG-CoA reductase, thereby reducing endogenous cholesterol production [56], whereas testosterone deficiency downregulates nuclear receptors such as LXR and PPAR-γ, both key modulators of lipid homeostasis [57]. Because many breast and prostate cancer treatments rely on pharmacological suppression or modulation of oestrogen or testosterone signalling, hormone-targeting therapies exert heterogeneous effects on lipid metabolism and CV risk [58]. Indeed, both oestrogen and testosterone deficiencies are independently associated with adverse lipid profiles, including rises in LDL, TC, and TG [59].

#### 5.8.1 Selective Estrogen Receptor Modulators

Selective estrogen receptor modulators (SERMs), such as tamoxifen, act as estrogen antagonists in tumour tissue but behave as partial agonists in the liver and other organs, producing a net favourable effect on lipid metabolism [60]. This estrogen-mimetic activity can attenuate chemotherapy-induced dyslipidaemia [61]. In clinical studies, tamoxifen has been associated with reductions in TC and LDL [62], although such improvements tend to reverse within six months of discontinuation [63]. Reports on TG changes are inconsistent, with some studies documenting elevations [64] and others showing neutral effects [64]. Beyond lipid effects, tamoxifen appears to confer CV protection: meta-analyses indicate a 25–35% reduction in CV events compared with placebo [65,66]. However, this benefit is not universal, and tamoxifen is associated with increased venous thromboembolism risk—a confounder in studies assessing CV outcomes [67].

#### 5.8.2 Aromatase Inhibitors

Aromatase inhibitors (AIs) reduce circulating oestrogen by blocking its peripheral synthesis [67]. Unlike SERMs, they lack hepatic oestrogen-mimetic activity and are consistently linked to higher CV risk [67]. In a large UK cohort, initiation of an AI rather than tamoxifen has been associated with a 1.5-fold increase in CV events after risk adjustment. Meta-analytic data similarly show increased CV risk with extended (≥5 years) AI therapy [67]. 

#### 5.8.3 Androgen Deprivation Therapy

Androgen deprivation therapy (ADT) is a cornerstone of advanced prostate cancer management [68,69]. ADT typically induces a characteristic dyslipidaemic profile—elevations in LDL, HDL, TG, and TC [70]—alongside increased arterial stiffness and insulin resistance [71]. 

### 5.9 Immunotherapy

Data on how immunotherapeutic agents affect lipid metabolism remain limited. Novel cellular therapies—such as chimeric antigen receptor T cells (CAR-T)—are increasingly used, particularly in hematologic malignancies, yet their impact on lipid pathways has not been defined. In contrast, immune checkpoint inhibitors (ICIs) have been extensively adopted across many solid and hematologic cancers and are well recognized for their broad spectrum of cardiometabolic toxicities [72].

Dysregulated Programmed Cell Death Protein 1 (PD-1)/Programmed Death Ligand 1 (PD-L1) signaling, as occurs with ICIs therapy, appears to promote atherosclerotic plaque progression and destabilization through alterations in intraplaque T-cell-mediated immune responses. In addition, interference with key regulatory pathways in cardiomyocytes has been implicated in myocarditis, vasculitis, accelerated atherosclerosis, arrhythmias, and pericardial disease [72].

A large cohort study of 2842 cancer patients treated with ICIs demonstrated a three-fold increase in atherosclerosis-related CV events [73]. In the same population, the incidence of CV events rose from 1.37 per 100 person-years pre-ICI to 6.55 per 100 person-years post-ICI. Moreover, in a dedicated imaging substudy of 40 individuals, atherosclerotic plaque volume increased three-fold following ICI exposure [73].

These findings suggest that ICIs may substantially amplify ASCVD risk, although their direct influence on lipid metabolism itself remains incompletely understood. A key clinical question is whether patients receiving ICIs should undergo more aggressive management of traditional cardiovascular risk factors, given their heightened vulnerability to adverse cardiac events [73].

**Table 1:** Effects of anticancer therapies on lipid metabolism and ASCVD risk.

Therapy Class	Main Mechanisms Affecting Lipid Metabolism	Typical Lipid Changes	Potential ASCVD Impact
Anthracyclines^1^	↓ LXR^1^/PPAR-γ activity; ↓ ABCA1-mediated cholesterol efflux; intracellular cholesterol accumulation	↑ TC, ↑ LDL, ↑ TG; ↓ HDL (variable)	↑ ASCVD risk; persistent dyslipidaemia reported
Taxanes	↑ HMG-CoAR activity; ↓ LDL receptor expression; altered apoB metabolism	↑ TC, ↑ LDL, ↑ TG, HDL variable	Likely additive/synergistic ASCVD risk
TKIs	Drug-specific effects on LDL trafficking, inflammation, and lipid handling	Variable: ↓ or ↑ cholesterol depending on TKI	Heterogeneous; requires individualized assessment
mTOR inhibitors	↓ LDL receptor expression; altered TG handling; ↓ lipogenic enzymes	↑ TC, ↑ TG; frequent hypercholesterolaemia	Net ASCVD risk uncertain; confounded by statin use
Alkylating agents	Minimal direct effect; dose-dependent metabolic changes	Generally neutral; dyslipidaemia mainly in combination regimens	No clear independent ASCVD risk
Platinum compounds	Possible delayed lipid alterations (mechanism unclear)	Inconsistent. Transient ↑ LDL/TG reported	Unclear; likely regimen-dependent
Antimetabolites	Possible ↑ cholesterol efflux	Variable; occasional ↑ TG (capecitabine)	Uncertain; drug-specific effects
SERMs (tamoxifen)	Hepatic oestrogen agonism; ↓ HMG-CoA reductase	↓ TC ↓ LDL; TG variable	↓ CV events; ↑ VTE risk
Aromatase inhibitors	Oestrogen depletion; loss of hepatic lipid protection	↑ LDL, ↑ TC	↑ ASCVD risk
Androgen deprivation therapy	Testosterone depletion; ↓ LXR/PPAR-γ signalling; insulin resistance	↑ LDL, ↑ HDL, ↑ TG, ↑ TC	↑ ASCVD risk
ICIs	Immune-mediated plaque inflammation; T-cell dysregulation	Lipid changes unclear	Marked ↑ ASCVD events

*Abbreviations: ↑: increase, ↓: reduction, ABCA1, ATP binding cassette transporter A1, ADT: androgen deprivation therapy, apoB: apolipoprotein B, ASCVD: atherosclerotic cardiovascular disease, CV: cardiovascular, HDL: high-density lipoprotein, HMG-CoA: 3-hydroxy-3-methylglutaryl-coenzyme A, ICI: immune checkpoint inhibitor, LDL: low-density lipoprotein, LDLR: low-density lipoprotein receptor, LXR: liver X receptor, mTOR: mechanistic Target of Rapamycin, PPAR-γ: peroxisome proliferator-activated receptor gamma, RNA: ribonucleic acid, ROS: reactive oxygen species, SERMs: selective oestrogen receptor modulator, TC: total cholesterol, TG: triglycerides, TKI: tyrosine kinase inhibitor, VTE: venous thromboembolism.

## 6 Lipid-Lowering Therapies in Patients with Cancer

**Figure 3:**
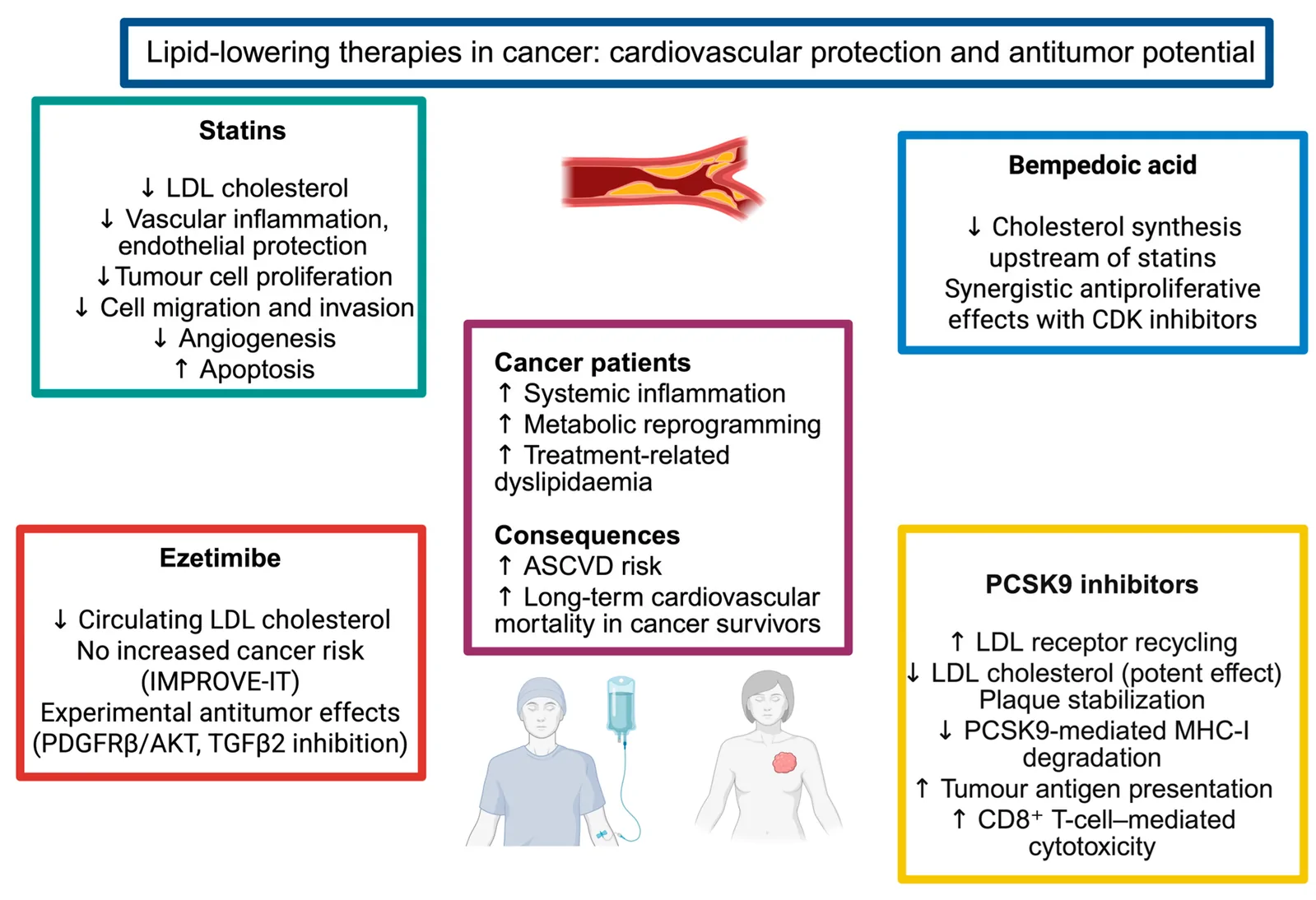
This figure summarizes the rationale and mechanistic basis for lipid-lowering therapies in oncology patients, integrating cardiovascular prevention with potential antitumour effects (Created in BioRender.com). Abbreviations: ASCVD: atherosclerotic CV disease, CD8^+^: cluster of differentiation 8-positive T lymphocytes, CDK: cyclin-dependent kinase, MHC-I: major histocompatibility complex class I, PCSK9: proprotein convertase subtilisin/kexin type 9, PDGFRβ: platelet-derived growth factor receptor beta, TGFβ2: transforming growth factor β2.

The importance of lipid-lowering therapies in patients with cancer is emphasized by the most recent European guidelines [74,75]. In the 2022 European Society of Cardiology (ESC) guidelines on cardio-oncology, hypercholesterolaemia is considered a major CV risk factor for patients scheduled to receive anticancer therapies [74]. Furthermore, the 2025 focused update of the ESC guidelines on dyslipidaemias reiterates that, regardless of baseline cholesterol levels, statin therapy should be considered to reduce the risk of anthracycline-induced cardiac dysfunction in adult cancer patients at high or very high risk of CV toxicity [75]. Furthermore, it is strongly advised an early combination lipid-lowering strategy, particularly in patients after acute myocardial infarction, in order to achieve rapid and sustained LDL-cholesterol targets. In this context, the addition of non-statin agents such as ezetimibe should be considered early rather than as a late step-up approach [75]. Despite these recommendations, a recent Italian survey showed that more than 30% of physicians did not prescribe adequate statin doses, mainly due to concerns about potential drug-drug interactions with anticancer therapies, despite the high estimated prevalence of dyslipidaemia in this population (ranging from 20% to 60%) [76] [Fig. 3].

### 6.1 Statins

Statins remain the most widely used and extensively studied lipid-lowering agents in oncology patients [77]. Beyond their established role in reducing LDL cholesterol and atherosclerotic CV disease risk, increasing evidence suggests that these drugs may exert direct anticancer effects by modulating tumour cell metabolism, signalling, and interactions within the microenvironment [77]. At the molecular level, these effects are primarily mediated through inhibition of the mevalonate pathway, a central metabolic axis linking cholesterol biosynthesis to multiple oncogenic processes [77]. By blocking HMG-CoAR, statins reduce intracellular levels of mevalonate-derived isoprenoid intermediates, including farnesyl pyrophosphate and geranylgeranyl pyrophosphate. These intermediates are essential for the post-translational prenylation of small GTP-binding proteins such as Rat sarcoma (Ras), Ras homologous (Rho), and Ras-related *C3 botulinum toxin substrate* (Rac) [78]. Impaired prenylation disrupts membrane localization and signalling activity of these proteins, resulting in attenuation of proliferative, migratory, and survival pathways that are frequently dysregulated in malignant cells [79].

In parallel, statin-induced cholesterol depletion alters the composition and biophysical properties of cellular membranes, particularly cholesterol-rich lipid rafts that serve as critical platforms for oncogenic signalling [79]. Disruption of lipid raft integrity impairs receptor clustering and downstream activation of pathways such as PI3K/AKT, and receptor tyrosine kinase signalling, thereby sensitizing tumour cells to apoptotic stimuli and cytotoxic therapies [78]. Consistent with these mechanisms, preclinical studies have demonstrated that statins inhibit tumour cell adhesion, migration, and invasion, suppress angiogenesis, and promote apoptosis across multiple cancer models [77]. Additional pleiotropic effects—including antioxidant, anti-inflammatory, immunomodulatory, and endothelial-protective actions—further contribute to modulation of the tumour microenvironment, limiting chronic inflammation, vascular remodelling, and tumour-associated angiogenesis [79].

Emerging data also indicate that statins may influence antitumour immunity by reshaping cholesterol metabolism within immune cells [80]. Alterations in intracellular cholesterol availability can modulate T-cell activation, differentiation, and exhaustion, potentially enhancing cytotoxic immune responses [80]. Moreover, these drugs have been shown to attenuate the immunosuppressive phenotype of tumour-associated macrophages and to interfere with myeloid-derived suppressor cell function, thereby fostering a more immune-permissive tumour microenvironment [80]. These combined effects support a biologically plausible role for statins as adjunctive modulators of tumour progression rather than purely lipid-lowering agents [81].

Consistent with these mechanistic insights, observational studies have reported associations between statin use and reduced incidence, progression, or cancer-specific mortality in several malignancies, including colorectal, pancreatic, breast, and lung cancers [80,81]. However, these findings are largely derived from observational cohorts and remain susceptible to confounding and indication bias, underscoring the need for large-scale prospective trials specifically designed to evaluate oncologic endpoints [81].

From a clinical perspective, selection of statin therapy in oncology patients requires careful consideration of drug-drug interactions and hepatic function. Simvastatin, lovastatin, and atorvastatin are predominantly metabolized by cytochrome P450 3A4 (CYP3A4), an enzyme frequently inhibited or induced by anticancer agents, particularly tyrosine kinase inhibitors [79,82]. Concomitant administration with CYP3A4 inhibitors such as nilotinib or ribociclib may increase statin plasma concentrations and heighten the risk of hepatotoxicity and rhabdomyolysis, whereas CYP3A4 inducers, including lorlatinib and pexidartinib, may reduce lipid-lowering efficacy [79]. In these settings, statins with more favourable pharmacokinetic profiles—such as rosuvastatin and pravastatin—are generally preferred. Similarly, in patients with hepatic impairment, rosuvastatin, pravastatin, and pitavastatin, which undergo minimal hepatic metabolism, represent safer options for lipid management in the oncology setting [79].

### 6.2 Ezetimibe

Ezetimibe, which inhibits intestinal cholesterol absorption, is the second most commonly used lipid-lowering agent after statins. In the past, concerns were raised regarding a potential increased risk of intestinal cancer associated with ezetimibe therapy; however, the IMPROVE-IT trial provided reassuring evidence showing no association between ezetimibe use and increased tumour risk [80]. Conversely, emerging evidence suggests a potential role for ezetimibe in the treatment of triple-negative breast cancer. Preclinical data indicate that ezetimibe can inhibit cell proliferation by blocking activation of the platelet-derived growth factor subunit β (PDGFRβ)/AKT signaling pathway and can suppress cancer cell migration, invasion, and epithelial-mesenchymal transition by significantly downregulating transforming growth factor β2 (TGFβ2) expression [83,84].

### 6.3 Bempedoic Acid

Data on the use of bempedoic acid in oncology patients remain limited. Preclinical studies have shown that inhibition of ATP-citrate lyase with bempedoic acid, in combination with palbociclib (a cell-cycle inhibitor), reduces proliferative activity in panels of breast and pancreatic cancer cell lines. These findings suggest a potential synergistic effect of the two agents, with possible therapeutic benefits in selected solid tumours [85]. Bempedoic acid has a favorable safety profile and fewer drug-drug interactions than statins, making it an attractive option in oncology patients. Its main adverse effect is dose-dependent hyperuricemia, which warrants careful monitoring in this population [85].

### 6.4 PCSK9 Inhibitors

PCSK9 inhibitors, primarily known for their potent lipid-lowering and plaque-stabilizing effects, are still infrequently used to treat dyslipidemia in oncology patients [84]. However, a large retrospective study involving more than 60,000 patients recently demonstrated that PCSK9i use was associated with a lower risk of all-cause mortality, including both CV and cancer-related mortality, among long-term cancer survivors [86].

Preclinical evidence suggests a direct involvement of PCSK9 in tumour biology. PCSK9 overexpression, which has been associated with worse prognosis, has been observed in several malignancies, including lung, breast, gastric, and colorectal cancers, hepatitis B virus-related hepatocellular carcinoma, and acute lymphoblastic leukemia [87]. PCSK9 appears to mediate immune evasion mechanisms in cancer cells—through degradation of major histocompatibility complex class I (MHC-I) molecules—and to promote cellular proliferation via activation of oncogenic signaling pathways, particularly the PI3K/AKT and Wnt/β-catenin cascades [86,87].

Several preclinical studies have shown that PCSK9 inhibition enhances the response to immunotherapy by increasing MHC-I expression and improving CD8^+^ T-cell-mediated cytotoxicity [88]. The combination of PCSK9 inhibitors with ICIs may therefore amplify antitumour efficacy while simultaneously mitigating systemic inflammation and accelerated atherosclerosis associated with ICIs therapy [89]. Moreover, in patients receiving ICIs who are at increased risk of myositis, statins should be avoided when possible, and PCSK9 inhibitors may represent a safer alternative for hypercholesterolemia management [90]. Currently, randomized clinical trials are ongoing to investigate the use of these drugs in oncology, in combination with ICIs in patients with non-small-cell lung cancer or with chemotherapy in patients with advanced pancreatic adenocarcinoma [91].

The dual lipid-lowering and antitumour effects of PCSK9 inhibitors make this drug class a promising candidate for the integrated cardiovascular management of oncology patients, with potential antitumor implications that remain under active investigation [92,93]. Their ability to modulate immune responses, in addition to finely regulating lipid metabolism, positions PCSK9 inhibitors as valuable agents within cardio-oncology pathways, especially in patients undergoing immunotherapy or those with refractory dyslipidemia [93]. Further studies are needed to fully elucidate the direct oncologic role of PCSK9 and to validate new therapeutic indications.

### 6.5 HDL Enhancing Therapies

Several HDL-targeted therapies, including Cholesteryl Ester Transfer Protein (CETP) inhibitors, synthetic HDL particles, and niacin, have been investigated to improve HDL levels or function [94]. However, despite their biological rationale, these strategies have not consistently demonstrated CV benefit in clinical trials and remain limited in clinical practice. As a result, current guidelines continue to prioritize LDL-cholesterol reduction over HDL-targeted interventions [74,75].

### 6.6 New Therapies

In the cardio-oncology context, cancer therapies characterized by increased oxidative stress and immune activation may amplify oxLDL generation, simultaneously accelerating atherosclerosis and fostering a pro-tumorigenic microenvironment [22]. OxLDL-LOX-1 signalling has also been implicated in immune dysregulation, influencing macrophage polarization, impairing dendritic cell function, and promoting T-cell dysfunction—mechanisms that may intersect with resistance to immunotherapy [22]. These observations position oxLDL not only as biomarkers of cardiometabolic risk but also as potential therapeutic targets [22]. Accordingly, pharmacological strategies aimed at modulating oxLDL pathways—including lipid-lowering agents, anti-inflammatory compounds, autophagy modulators, and emerging LDL-mimetic nanoparticle platforms—are being explored for their dual capacity to mitigate CV toxicity and interfere with tumour metabolism and angiogenesis [22]. Targeting oxLDL-driven pathways may therefore represent a promising integrative strategy within cardio-oncology to reduce both cancer progression and treatment-related cardiovascular disease [22].

## 7 Conclusion

A growing body of evidence indicates that cholesterol metabolism plays a significant role in tumorigenesis and cancer progression, although many questions remain unanswered. The complex interplay between cancer, lipid metabolism, and atherosclerotic CV disease underscores the need for further targeted studies to evaluate the prognostic impact of lipid-lowering therapies in oncology patients, both to reduce CV risk and to exploit the potential antitumour properties of these agents.

The 2022 ESC cardio-oncology guidelines and the 2025 update of the dyslipidaemia guidelines emphasize the importance of statin therapy for the prevention of anthracycline-induced cardiotoxicity and highlight the need for optimization of lipid profiles in cancer patients, recognizing dyslipidaemia as a dominant risk factor for the development of cardiotoxicity [74,75]. The role of other lipid-lowering therapies in oncology patients remains largely confined to the management of resistant dyslipidaemia; however, emerging preclinical evidence is generating increasing interest in PCSK9 inhibitors as potential antineoplastic agents.

In this context, cardio-oncology plays a strategic role in the integrated management of metabolic and cardiovascular risk in patients with cancer. It represents a key discipline for coordinated clinical care and for guiding and promoting the development of novel therapeutic strategies.

## Data Availability

This statement should make clear how readers can access the data used in the study and explain why any unavailable data cannot be released.

## Abbreviations

27HC	27-hydroxycholesterol
ABCA1	ATP binding cassette transporter A1
ADTs	Androgen deprivation therapy
AIs	Aromatase inhibitors
apoB	Apolipoprotein B
AKT	Protein Kinase B
ASCVD	atherosclerotic cardiovascular disease
ATPG5	ATP binding cassette transporter G5
ATPG8	ATP binding cassette transporter G8
ATPA1	ATP binding cassette transporter A1
ATPG1	ATP binding cassette transporter G1
BCR-ABL1	Breakpoint Cluster Region Abelson murine leukaemia viral oncogene homolog 1
CV	Cardiovascular
CAR-T	Chimeric antigen receptor T cells
CD36	Cluster of differentiation 36
CYP3A4	Cytochrome P450 3A4
CMLs	Chylomicrons
DNA	Deoxyribonucleic acid
ESC	European Society of Cardiology
GDP	Guanosine diphosphate
HDL	High density lipoprotein
HMG-CoA	3-hydroxy-3-methylglutaryl-coenzyme A
HMG-CoAR	3-hydroxy-3-methylglutaryl-coenzyme A reductase
ICIs	Immune checkpoints inhibitors
IDL	Intermediate lipoprotein density
LDL	Low-density lipoprotein
LDLR	LDL receptor
LOX-1	Lectin-like oxidized LDL receptor-1
LPL	Lipoprotein lipase
LXR	Lipoprotein X receptor
MHC1	Major histocompatibility complex I
mTOR	Mechanistic target of rapamycin
NF-kB	Nuclear Factor kappa-light-chain-enhancer of activated B cells
NPC1L1	Niemann-Pick C1-like 1
oxLDL	Oxidized LDL
PCSK9	Proprotein Convertase Subtilisin/Kexin type 9
PDL-1	Programmed death ligand 1
PD1	Programmed Cell Death Protein 1
PI3K	Phosphatidylinositol 3-kinase
PPARγ	Peroxisome Proliferator-Activated Receptor γ
Rac	Ras-related C3 botulinum toxin substrate
Ras	Rat sarcome
Rho	Ras homologous
RNA	Ribonucleic acid
ROS	Reactive oxygen species
SERMs	Selective estrogen receptor modulators
SREBPs	Sterol regulatory element-binding proteins
TC	Total cholesterol
TG	Triglycerides
TGFβ2	Transforming Growth Factor β2
TKIs	Tyrosine kinase inhibitors
VLDL	Very low density lipoprotein
Wnt	Wingless-related integration site
